# Impact of Frailty on Symptom Burden in Chronic Obstructive Pulmonary Disease

**DOI:** 10.3390/jcm13040984

**Published:** 2024-02-08

**Authors:** Alessia Verduri, Enrico Clini, Ben Carter, Jonathan Hewitt

**Affiliations:** 1 Department of Population Medicine, Cardiff University, Cardiff CF14 4XN, UK; 2 Respiratory Unit, Department of Surgical and Medical Sciences, University of Modena and Reggio Emilia, 41124 Modena, Italy; 3 Department of Biostatistics and Health Informatics, Institute of Psychiatry, Psychology and Neuroscience, King’s College London, London SE5 8AB, UK

**Keywords:** COPD, frailty, symptoms, dyspnoea, GOLD guidelines, mMRC scale, COPD Assessment Test

## Abstract

Chronic obstructive pulmonary disease (COPD), the sixth leading cause of death in the United States in 2022 and the third leading cause of death in England and Wales in 2022, is associated with high symptom burden, particularly dyspnoea. Frailty is a complex clinical syndrome associated with an increased vulnerability to adverse health outcomes. The aim of this review was to explore the current evidence of the influence of frailty on symptoms in patients with a confirmed diagnosis of COPD according to GOLD guidelines. Fourteen studies report a positive association between frailty and symptoms, including dyspnoea, assessed with the COPD Assessment Test (CAT) and the modified Medical Research Council (mMRC) scale. Data were analysed in a pooled a random-effects meta-analysis of mean differences (MDs). There was an association between COPD patients living with frailty and increased CAT score versus COPD patients without frailty [pooled SMD, 1.79 (95% CI 0.72–2.87); *I*^2^ = 99%]. A lower association was found between frailty and dyspnoea measured by the mMRC scale versus COPD patients without frailty [pooled SMD, 1.91 (95% CI 1.15–2.66); *I*^2^ = 98%]. The prevalence of frailty ranged from 8.8% to 82% and that of pre-frailty from 30.4% to 73.7% in people living with COPD. The available evidence supports the role of frailty in worsening symptom burden in COPD patients living with frailty. The review shows that frailty is common in patients with COPD. Future research is needed to have further details related to the data from CAT to improve our knowledge of the frailty impact in this population.

## 1. Introduction

Chronic obstructive pulmonary disease (COPD) is one of the leading causes of morbidity and remains one of the top three causes of death worldwide [1]. The global prevalence of COPD increases with age, and the burden related to is expected to rise over the next years due to persistent exposure to risk factors and ageing of the world’s population [1,2]. According to the GOLD criteria, roughly 400 million people have been reported to suffer from COPD globally in 2019 [3]. COPD is characterised by chronic respiratory symptoms, including dyspnoea and activity limitation, that have a negative impact on patients’ quality of life and increase the risk of depression and anxiety [1].

Frailty is a complex syndrome described as a state of reduced physiological reserve causing increased vulnerability to adverse events and external stressors. This vulnerability can result in disability, admission to a hospital or a long-term facility, and increased risk of all-cause mortality. There are different validated tools available to measure frailty, ranging from simple assessment of functional status to complex, multi-domain scoring systems that evaluate cognitive, physical, and functional ability [4,5,6,7]. Two main classifications of frailty (the deficit model and the phenotype model) are widely accepted and frequently used in clinical practice [5,8]. The frailty phenotype model proposed by Fried et al. [5] is defined as the presence of three or more of the following domains: exhaustion, weakness, slowness, low activity, and unintentional weight loss. The frailty index (FI) [8] is a quantification of the cumulative burden of health deficits including laboratory findings, physical function disabilities, diseases, symptoms, sensory difficulties, and cognition difficulties.

The prevalence of frailty in COPD varies according to the criteria of the frailty model used, ranging between 6.4 and 72% [9]. In this review, we examine the association between frailty and symptom burden, including dyspnoea, in patients with COPD in the most recent accumulated evidence. By summarising the available evidence from the included studies, this review also aims to identify future directions for research and clinical practice in this field.

## 2. Overview of Symptoms in COPD

*Dyspnoea*, also known as shortness of breath or breathlessness, is the most typical symptom and debilitating for patients with COPD and is associated with a deterioration in their physical activity [10]. Dyspnoea is defined as “a subjective experience of breathing discomfort that consists of qualitatively distinct sensation that vary in intensity” [11] and results from multiple mechanism, including air trapping, dynamic hyperinflation, and gas exchange abnormalities [12]. *Fatigue*, described as an individual feeling of tiredness and being drained of energy until exhaustion, is one of the most common symptoms in COPD. It has an impact on activities of daily living and is associated with frustration and problems with concentration [13]. The intensity of fatigue is variable and, similar to dyspnoea, can be increased by intercurrent exacerbation of COPD. Other cardinal symptoms of patients with COPD include *chronic cough with sputum production, chest tightness*, and *wheezing* which may be recurrent as well [1].

The prevalence of dyspnoea is high across all stages of airflow obstruction [14]. Dyspnoea intensity has been associated with age ≥70 years, higher severity of airflow obstruction, female gender, obesity, history of moderate-to-severe exacerbations, and comorbidities [15]. Fatigue has been also reported in mild COPD [16].

## 3. Assessment of Symptoms in COPD

The degree of airflow obstruction can be a poor effective predictor of symptom burden in COPD, especially for dyspnoea and fatigue. Validated questionnaires have been developed to promote a more comprehensive evaluation of breathlessness and other relevant symptoms in these patients. All symptoms in COPD represent an individual experience, difficult to quantify numerically with questionnaires; hence, several scales are available both in research settings and clinical practice [1].

**Figure 1:**
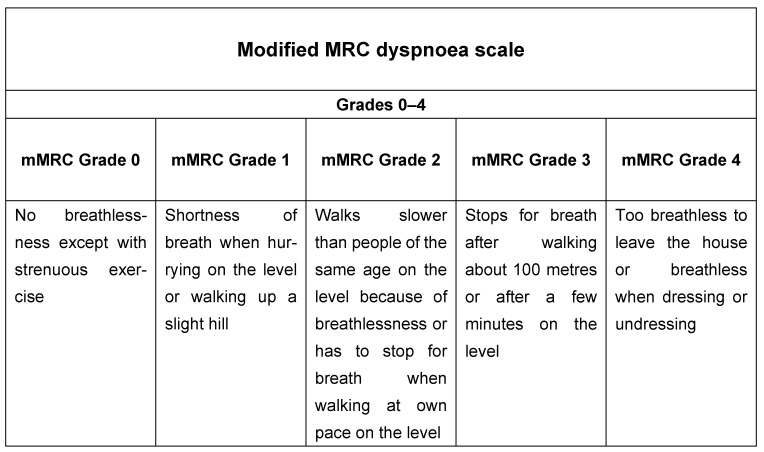
Modified Medical Research Council dyspnoea scale [17].

**Figure 2:**
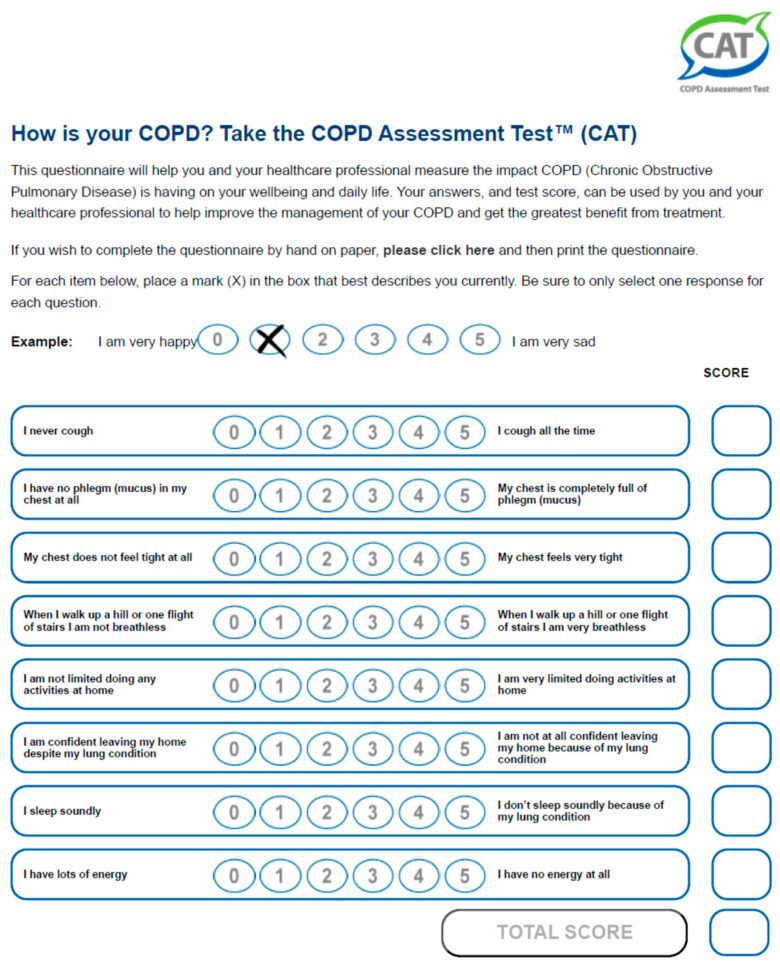
COPD Assessment Test.

These include the modified Medical Research Council (mMRC) dyspnoea scale (Figure 1), including five statements to measure the degree of disability due to breathlessness in daily activities on a scale from 0 to 4 [17,18], and multidimensional questionnaires such as the COPD Assessment Test (CAT) (Figure 2), which includes eight items describing the clinical manifestations of COPD. Cough, presence of mucus, chest tightness, dyspnoea, limitation in activities of daily living, feeling confident, sleeping, and energy are the components of the test, scoring 0 to 40 [19]. Both are used to describe level of symptoms in the Global Initiative for Chronic Obstructive Lung Disease (GOLD) recommendations that suggest a therapeutic strategy based on the history of exacerbations and burden of symptoms in stable COPD (GOLD 2023) [1].

## 4. Psychological Burden of Symptoms in COPD Patients

Dyspnoea and fatigue have been associated with anxiety and depression in COPD [20]. These comorbidities may result from the distress related to breathlessness and fatigue that are perceived as negative emotions associated with fear and feeling of sadness by patients [11], resulting in worse patient health and quality of life. Whilst these comorbidities have not been fully studied, the reported prevalences, mainly due to the population assessed in primary or secondary care settings, vary widely with mean range of 35–40% for depression and 28–36% for anxiety [21,22]. Anxiety and fear related to COPD are prevalent complications that widely contribute to disability and difficulties in activities of daily living in this population. Many patients adopt a sedentary lifestyle in anticipation of dyspnoea, and fear of exhaustion can result in experiencing more severe shortness of breath during exercise [23]. It has been noted that patients with COPD and associated depression experience more dyspnoea than their counterparts without depression [24]. The depression symptoms may range from sub-clinical depressive symptoms to clinical depression and, along with anxiety, have an effect on lack of energy, irritability, and feelings of demoralisation [23,25].

## 5. Frailty in COPD

A Task Force of the European Respiratory Society (ERS) has recently published a report on frailty in chronic lung diseases, including COPD [26]. The report has highlighted that frailty assessment leads to a more personalised management of patients with COPD, covering different aspects such as nutrition and rehabilitation. Apart from Australia and New Zealand Guidelines [27], frailty has not been considered in the guidelines of COPD up to 2022, despite relevant data on its prevalence and influence in morbidity and mortality related to COPD. For the first time, the 2023 GOLD Report mentioned a large observational study on the prevalence of frailty in people with COPD compared to individuals without COPD, showing a higher risk of poor outcomes [28].

## 6. Frailty Prevalence in COPD

Frailty is common in COPD. This has been confirmed by several studies [9,29,30]. The prevalence of frailty may vary according to the model used and the setting in which a population is from [31]. The following instruments are examples: Clinical Frailty Scale (CFS) [6], FRAIL Scale [32], Frailty Index-Comprehensive Geriatric Assessment (FI-CGA) [7], Canadian Study of Health and Aging Clinical Frailty Scale (CSHA-CFS) [33], FiND questionnaire [34], PRISMA-7 questionnaire [35]. Generally, frailty is measured as a binary variable (either frail or not frail) using the thresholds presented for each individual instrument (Table 1).

With the use of the phenotype model and the deficit model, frailty prevalence was 17% and 32%, respectively [29]. The prevalence varied from 9% to 64% according to the Fried criteria and from 9% to 28% when different frailty models were used [30]. In COPD patients with a diagnosis according to GOLD recommendations, the median prevalence of frailty was nearly 50%, ranging from 6.4% to 72% [9]. Similar data described by Wang et al. showed an overall prevalence of 32% (range 6.4 % to 71.7%) [36].

## 7. Frailty and Symptoms in COPD

Whilst this review is not a systematic review, evidence was based on original articles obtained through a comprehensive search in October 2023 and limited to publications posted in the last 15 years in four databases (PubMed, Web of Science, The Cochrane Library, and EMBASE), using the Medical Subject Headings (MeSH) terms “Frailty”, “Dyspnoea”, “Symptoms”, and “COPD” as keywords. Only English language manuscripts with full-text availability were considered. Exclusion criteria were as follows: not original research articles, patients with diagnosis of COPD not based on the guidelines [1], lack of a validated method of frailty identification.

The link between frailty and symptoms in COPD was explored in 14 [37,38,39,40,41,42,43,44,45,46,47,48,49,50] original research articles across 3097 participants (50% male, 1563/3097) with available data for comparison (Table 2). Of the 14 studies, published between 2016 and 2021, 6 were cohort studies and 8 were cross-sectional. Thirteen studies were conducted among outpatients with a diagnosis of COPD according to GOLD guidelines. One study included community-dwelling adults in the primary care setting; likewise, COPD diagnosis was based on GOLD guidelines [43]. Only one study examined outpatients with severe COPD with chronic respiratory failure defined as the use of long-term oxygen therapy and/or non-invasive ventilation [49].

The vast majority of COPD participants in these studies were older people (overall mean age 70.3). Three studies did not report any age cut-off or age range [39,43,48], but the mean age of individuals was between 65 and 73 (Gale mean age 66.1, Finamore mean age 73, Ierodiakonou mean age 65). Whilst the range of ages may have included younger participants, the patients with COPD examined were representative of older people.

Seven different and validated tools [Fried Frailty Phenotype, Frailty Index-Comprehensive Geriatric Assessment (FI-CGA), Frailty Index, FRAIL Scale, FiND questionnaire, PRISMA-7 questionnaire, Kihon Checklist] [5,7,8,32,34,35,51] were utilised to assess frailty in these studies and considered suitable for inclusion in the estimation of frailty prevalence in COPD. The phenotype model [5], the deficit model [8], and the Kihon Checklist [51] were the most commonly used. Apart from the FRAIL scale [32] and PRISMA-7 questionnaire [35] which are self-administered, all other instruments are conducted by a healthcare professional.

The most used dyspnoea scale and symptom questionnaires were the mMRC and the CAT, respectively. Seven studies used both mMRC and CAT. Twelve studies [37,38,39,40,41,42,43,44,45,46,47,50] found a positive association between frailty and dyspnoea, whilst two studies reported no association [48,49].

Seven studies [37,38,40,44,46,47,50] divided patients into three groups: Non-Frail, Frail, and Pre-Frail. The remaining seven [39,41,42,43,45,48,49] classified patients into two groups: Non-Frail and Frail.

Two further studies have been considered by this review, but not included in the meta-analysis due to study design and lack of raw data. One study [52] divided COPD patients into two groups: a non-dyspnoea group (mMRC 0–1) and a dyspnoea group (mMRC ≥ 2). A positive association was described between frailty (using the CSHA-CFS), and the CAT score (*p* < 0.001), and the prevalence of frailty was higher in the dyspnoea group (*p* = 0.001). The second study [53] found a positive association between frailty and mMRC dyspnoea grade (*p* < 0.001) and between frailty status and CAT score (*p* < 0.001).

### Data Analysis

Primary outcomes were narratively described. Studies deemed as clinically and contextually homogeneous were considered for pooling, and they were associated with frailty as a binary variable based on the classification of subjects in two groups: non-frail and frail (including both frail and pre-frail individuals).

Where the included studies provided continuous outcome data as median and interquartile range (IQR), the median and IQR were converted to mean and standard deviation [54,55]. Homogeneous studies were pooled using an inverse variance method with random effects. Pooled effects were presented as standard mean differences (SMDs) with associated 95% CIs, *p*-values, and *I*^2^ summary data. All pooled meta-analyses were performed using Review Manager Version 5.4.

Statistical heterogeneity was measured using the *I*^2^ statistic. Heterogeneity exceeding 80% was explored using subgroup analyses. Pre-specified subgroups for exploring heterogeneity included age, gender, study design, type of frailty instrument, and study level risk of bias.

## 8. Results

**Figure 3:**
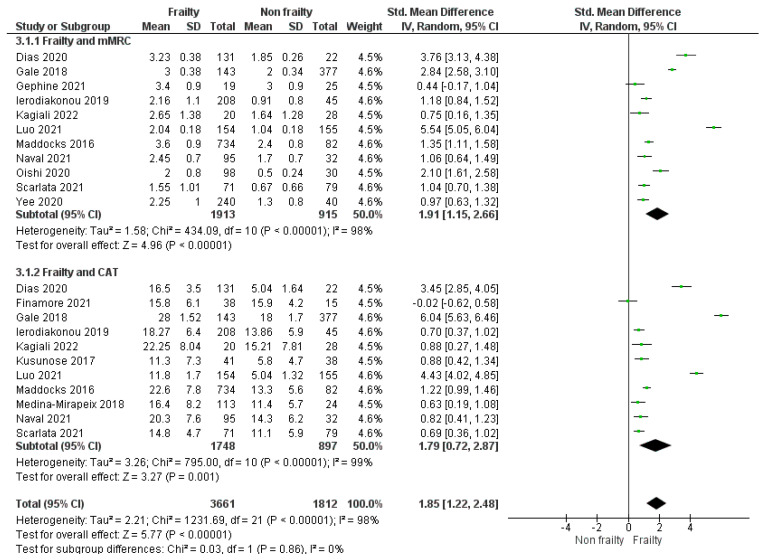
Impact of frailty on mMRC grade and CAT score in patients with COPD living with frailty compared to COPD patients without frailty.

Data from 11 studies [37,38,39,40,41,42,43,44,45,47,49] with 2828 participants were available for a meta-analysis of mMRC grades, showing that COPD patients living with frailty or pre-frailty had a higher dyspnoea score compared to non-frail COPD patients [SMD 1.91 (95% CI 1.15–2.66); *I*^2^ = 98%] (Figure 3). Data from 11 studies [37,38,39,41,42,43,45,46,47,48,50] with 2645 individuals were available for a meta-analysis of CAT scores, showing that COPD patients living with frailty or pre-frailty had a higher symptom score compared to non-frail COPD patients [SMD 1.79 (95% CI 0.72–2.87); *I*^2^ = 99%] (Figure 3). Considering the studies included in the meta-analysis, the mean CAT score was 18 (range 11.3–28) in the frail group and 11.7 (range 5.04–15.9) in the non-frail group, and the mean mMRC grade was 2.57 (range 2–3.6) in patients with frailty and 1.54 (range 0.5–3) in those without frailty. Overall, there was stronger association found with patients with COPD living with frailty and increased CAT score compared to non-frail COPD patients. A lower association was found between frailty and mMRC dyspnoea grade versus non-frail COPD patients.

Based on the different frailty tools, the prevalence of frailty in the 14 included studies [37,38,39,40,41,42,43,44,45,46,47,48,49,50] ranged from 8.8% to 82%, the prevalence of pre-frailty from 30.4% to 73.7% [37,38,40,44,46,47,50].

### Psychological Burden of Symptoms in COPD

The relationship between psychological disorders and chronic symptoms in COPD has been explored in one study [46] in which anxiety was a significant determinant of the CAT score in COPD, using the Hospital Anxiety and Depression Scale (HADS) [56]. A few of the studies included in this review showed contrasting findings related to the association between psychological comorbidities and frailty in COPD. Higher scores of anxiety and depression using the HADS in pre-frail and frail groups compared to robust COPD patients have been observed in three studies [37,47,49]. The depression score has been found to be strongly associated with frailty [47], and the presence of anxiety and depression has been related to the non-completion of a pulmonary rehabilitation programme [37]. By using the Kihon Checklist which includes depression as a domain and the HADS, one study found a strong correlation between the frailty score and anxiety and depression scores in patients with COPD [53]. By using the Center for Epidemiological Studies Depression Scale (CES-D scale) [57], in contrast, one study [52] did not find any significant difference in CES-D score between dyspnoea and non-dyspnoea groups in COPD, and no difference between non-frail and frail COPD patients was observed using the 15-item Geriatric Depression Scale [48,58].

## 9. Discussion

The main objective of this review was to explore the role of frailty in symptom burden in people with COPD.

Increased burden of symptoms in COPD, diagnosed using the GOLD guidelines, was associated with being frail considering both the CAT score and the mMRC scale [17,18,19]. The studies we considered for the review examined people with a confirmed diagnosis of COPD in an outpatient setting, the chronic disease object of study was COPD, and the frailty instruments used were all validated.

The positive association was stronger between the presence of frailty and the CAT score. This may be explained as the CAT is a more comprehensive assessment of symptoms of COPD, being specifically designed for COPD. The CAT is a multidimensional questionnaire able to capture the complexity of COPD and its impact on the daily life of patients better than the simple measurement of dyspnoea with the mMRC scale. The level of symptoms of a patient with COPD remains clinically relevant, even though the 2023 report of GOLD emphasised the importance of exacerbations. Relief of dyspnoea and fatigue during daily life activities has to be a major goal of COPD care to limit physical inactivity and disability in this group of patients. The mean CAT score in COPD patients living with frailty was nearly 20, indicating a great impact on their health, although COPD patients without frailty showed a mean score of 12 that is not unimportant. As reported in the guidelines, the CAT scores correlate with the GOLD strategy, which outlines an evidence-based plan for assessing and managing COPD, and if frailty is associated with the CAT score, this is clinically relevant.

The review also examined the prevalence of frailty in COPD. Based on the different frailty instruments in the included studies, the range of prevalence was between 9 and 82%. These data suggest that COPD as chronic disease can be considered a determinant of frailty. COPD and frailty share common risk factors such as age and smoking, and potentially chronic inflammation. Reduced physical activity is quite common in old age, irrespective of COPD. The presence of COPD leads to a more sedentary lifestyle, increasing the risk of frailty in older individuals with the disease. Different factors may contribute to frailty in COPD that are yet unexplored, for instance, the socio-economic status and the presence of anorexia. Poverty and low social support are both associated with a higher prevalence of COPD [59] and may potentially facilitate the occurrence of frailty. Weight loss, malnutrition, and anorexia are experienced by patients with COPD [60,61]. These aspects negatively contribute to muscle function and exhaustion and substantially to the appearance of frailty. All these factors, including sarcopenia, need to be evaluated in future studies on frailty in COPD.

A few of the tools used to measure frailty in the included studies also explore cognitive function. The presence of cognitive problems remains poorly understood in COPD, and further studies using multi-domain frailty instruments are needed to evaluate cognitive ability in people with COPD.

While there were a limited number of studies, they demonstrated a positive association between psychological disorders and the presence of frailty in COPD. Increased risk of depression and anxiety has been reported in patients with COPD and frailty [37,47,49,53], although two studies showed no association [48,52]. These findings are in keeping with the broader established association between frailty and depression [62].

### Limitations

This review had some limitations. The included studies that have been considered were only non-randomised studies, and reverse causality cannot be implied. There was also heterogeneity between studies, which may also impact conclusions.

## 10. Conclusions and Future Directions

This review was intended to provide a comprehensive analysis of the existing literature within the field of frailty and symptom burden in COPD. The findings show there is evidence of an association between the frailty status and symptoms detected by the CAT or the mMRC grade. These results are consistent with frailty adversely contributing to chronic symptoms in COPD. Although it is not possible to identify which answers to the CAT questionnaire were the determining factors in the final score, we can argue that dyspnoea, activities of daily living, and level of energy are real-life problems of patients with COPD. Limited studies also demonstrated an association between frailty, COPD, and depression.

A strength of this review is including studies with patients with a diagnosis of COPD defined using the international GOLD guidelines [1]. Therefore, this review provides by far the most accurate estimate of frailty with COPD and related data.

The available evidence indicates that further efforts are required to help people with COPD with novel management options. Frailty assessment should be considered in routine daily practice for all people living with COPD. The early identification of patients with COPD living with frailty may improve the management of their symptoms. Activity-related dyspnoea and fatigue and reduced physical activity are the key features of COPD management. Clinical interventions such as pulmonary rehabilitation may have a potential role in reversing the effect of frailty. Promoting the importance of physical activity will be beneficial in reducing the symptom-related impact on the quality of life of this patient group.

## Figures and Tables

**Table 1:** The frailty assessment tools used in the included studies.

References	Frailty Scale	Items Measured	Scoring	Administration
Fried et al., 2001	**Fried Frailty phenotype** (Physical Frailty Phenotype)	5 domains: Slowness Physical activity Weight loss Exhaustion Weakness	Score range: 0 to 5. Frail = ≥3 criteria Pre-frail = 1–2 criteria Non-frail = 0	Physician and self-reported
Mitnitski et al., 2001; Rockwood et al., 2007	**Frailty Index** (Deficit Accumulation Index)	Scales vary in content and number of items, generally 30–70. Multiple domains including laboratory findings, physical function disabilities, diseases, symptoms, sensory difficulties, cognition difficulties	Number of deficits present and divided by the number of deficits considered. Higher proportion = higher level of frailty.	Physician
Rockwood et al., 2011	**Frailty Index-Comprehensive Geriatric Assessment** (Modified for community-dwelling individuals)	Motivation, self-rated health, cognition, emotional, sleep, communication, strength, mobility, balance, elimination, nutrition, ADLs, IADLs, social engagement, medical history	Dividing the total number of CGA deficits by the maximum score of 61.	Physician
Abellan van Kan et al., 2008; Morley et al., 2012	**FRAIL Scale**	5 domains: Fatigue Resistance Ambulation Illnesses Loss of weight	Score range: 0 to 5. Non-frail = 0 deficits Intermediate frail = 1 or 2 deficits Frail = 3 or more deficits	Self-reported
Cesari et al., 2014	**Frail Non-Disabled (FiND) questionnaire**	5 questions: Disability: A difficulty at walking; B difficulty at climbing up Frailty: C weight loss; D limited activities; E level of physical activity	Disabled = A + B ≥ 1 Frail = A + B = 0 and C + D + E = ≥ 1 Non-frail = A + B + C + D + E = 0	Physician
Tomata et al., 2011	**Kihon Checklist**	25-item questionnaire including 7 domains: instrumental activity of daily living, social activity of daily living, physical strength, nutritional status, oral function, cognitive status, depression risk	Non-frail = 0–3 Pre-frail = 4–7 Frail = ≥8	Physician
Turner G et al., 2014; Raiche M et al., 2008	**PRISMA-7 questionnaire**	7 yes/no questions about the following: (1) Age; (2) Gender; (3) Health problems that limit activities; (4) Help needed from someone regularly; (5) Health problems that require staying at home; (6) Having someone to count on if needed; and (7) Regular use of an assistive device for walking.	Answering yes to three or more of the seven questions = potential disabilities/frailty	Self-reported

**Table 2:** The included studies for meta-analysis on frailty, mMRC grade, and CAT score in COPD.

Author, Year, Country	Patients, Study Design	Age ^£^	Frailty Scale	Frailty Prevalence in COPD (%)	Frailty Status (Non-Frail, Pre-Frail, Frail) in COPD (%)	Key Findings on mMRC Grade [mean ± SD or median (IQR)] and Frailty	Key Findings on CAT Score [mean ± SD or median (IQR)] and Frailty
Oishi K et al., 2020 Japan [44]	128 COPD outpatients in stable condition (cross-sectional study)	≥40 yrs 73 (69–78)	Kihon Checklist	48/128 (37.5%)	23.4%–39%–37.5%	The mMRC values were 3 (2–4) in frail COPD group, 1 (0–2) in pre-frail group, and 0.5 (0–1) in non-frail group. The higher the level of mMRC grade, the higher the proportion of frailty (*p* < 0.0001).	-
Kagiali S et al., 2021 Turkey [45]	48 COPD outpatients in stable condition (cross-sectional study)	>55 yrs 67.3 ± 5.1 Frail COPD patients 65.1 ± 4.6 Non-frail COPD patients	Fried Frailty Phenotype	20/48 (41.6%)	58.4%–NA–41.6%	The mMRC values were higher in frail COPD group (2.65 ± 1.38) versus non-frail COPD group (1.64 ± 1.28) (*p* = 0.018).	The CAT score was higher in frail COPD (22.25 ± 8.04) group compared to non-frail COPD group (15.21 ± 7.81) (*p* = 0.005).
Gephine S et al., 2021 Canada [49]	44 COPD outpatients with chronic respiratory failure in stable condition, starting pulmonary rehabilitation (prospective study)	≥40 yrs 66 ± 8	Fried Frailty Phenotype	19/44 (43%)	57%–NA–43%	The mMRC values were 3.4 ± 0.9 in frail COPD group and 3.0 ± 0.9 in non-frail COPD group: negative association between frailty and mMRC dyspnoea score (*p* = 0.07).	-
Dias LS et al., 2020 Brazil [38]	153 COPD outpatients in stable condition (cross-sectional study)	>40 yrs 68.8 (60.5–80.5)	FRAIL scale	77/153 (50.3%)	14.4%–35.3%–50.3%	The mMRC values were higher in frail COPD group [4 (2–4)] versus pre-frail group [2.5 (2–3)] and non-frail group [2 (1–2)] (*p* = 0.001 and *p* < 0.001; *p* = 0.03 between non-frail and pre-frail groups).	The CAT score was higher in frail COPD group [20 (13.5–26)] compared to pre-frail group [13 (7.8–19)] and non-frail group [5 (2–8.3)] (*p* = 0.001 and *p* < 0.001); *p* = 0.002 between non-frail and pre-frail groups).
Gale NS et al., 2018 United Kingdom [39]	520 COPD outpatients in stable condition (cross-sectional study) and 150 comparators	No age cut-off 66.1 ± 7.6	Frailty Index-CGA	143/520 COPD (27.5%)	72.5%–NA–27.5%	The mMRC values were higher in frail COPD group [3 (2–4)] versus non-frail COPD group [2 (1–3)] (*p* < 0.001).	The CAT score was higher in frail COPD group [28 (24–32)] versus non-frail COPD group [18 (13–23)] (*p* < 0.001).
Medina-Mirapeix F et al., 2018 Spain [46]	137 COPD outpatients in stable condition (cross-sectional study)	40-80 yrs 66.9 ± 8.3	Fried Frailty Phenotype	12/137 (8.8%)	17.5%–73.7%–8.8%	-	The CAT score was higher in frail COPD group (18.4 ± 9.3) versus pre-frail group (14.4 ± 7.2) and non-frail COPD group (11.4 ± 5.7) (*p* = 0.021).
Naval E et al., 2021 Spain [47]	127 COPD outpatients in stable condition (cross-sectional study)	>40 yrs 66.5 ± 7.9	Fried Frailty Phenotype	31/127 (24.4%)	25.2%–50.4%–24.4%	The mMRC values were higher in frail COPD group (2.9 ± 0.7) versus pre-frail group (2 ± 0.7) and non-frail group (1.7 ± 0.7) (*p* = 0.033).	The CAT score was higher in frail COPD group (23.4 ± 6.5) versus pre-frail group (17.2 ± 8.7) and non-frail COPD group (14.3 ± 6.2) (*p* = 0.002).
Finamore P et al., 2021 Italy [48]	53 COPD outpatients during and after pulmonary rehabilitation (prospective study)	No age cut-off 73 ± 8	PRISMA-7 Questionnaire	38/53 (72%)	28%–NA–72%	-	The CAT score was 15.8 ± 6.1 in frail COPD group and 15.9 ± 4.2 in non-frail group: negative association between frailty and CAT score (*p* = 0.91).
Yee N et al., 2020 United States [40]	280 COPD outpatients in stable condition (prospective study)	≥40 yrs 68 (mean)	Fried Frailty Phenotype	64/280 (23%)	14%–63%–23%	The mMRC values were higher in frail COPD group (2.6 ± 1) versus pre-frail group (1.9 ± 1.1) and non-frail group (1.3 ± 0.8) (*p* < 0.0167 between all groups).	-
Scarlata S et al., 2021 Italy [41]	150 COPD outpatients in stable condition (retrospective study)	≥40 yrs 73 ± 8	Frailty Index	71/150 (47.4%)	52.6%–NA–47.4%	The mMRC values were higher in frail COPD group (1.55 ± 1.01) versus non-frail group (0.67 ± 0.66) (*p* < 0.001).	The CAT score was higher in frail COPD group (14.8 ± 4.7) versus non-frail COPD group (11.1 ± 5.9) (*p* = 0.01).
Luo J et al., 2021 China [42]	309 COPD outpatients in stable condition (prospective study)	>65 yrs 86 (80–90)	Fried Frailty Phenotype	154/309 (49.8%)	50.2%–NA–49.8%	The mMRC values were higher in frail COPD group [2 (2–3)] versus non-frail COPD group [1 (1–2] (*p* < 0.001).	The CAT score was higher in frail COPD group [12 (6–15)] versus non-frail COPD group [5 (2–9)] (*p* < 0.001).
Maddocks M et al., 2016 United Kingdom [37]	816 COPD outpatients referred to pulmonary rehabilitation (prospective study)	>35 yrs 69.8 ± 9.7	Fried Frailty Phenotype	209/816 (25.6%)	10.1%–64.3%–25.6%	The mMRC values were higher in frail COPD group (4 ± 0.9) versus pre-frail (3.2 ± 1.0) and non-frail (2.4 ± 0.8) COPD group (*p* < 0.001).	The CAT score was higher in frail COPD group (25 ± 7.9) group compared to pre-frail (20.2 ± 7.8) and non-frail (13.3 ± 5.6) COPD group (*p* < 0.001).
Kusunose M et al., 2017 Japan [50]	79 COPD outpatients (cross-sectional study)	>50 yrs 74.8 ± 6.3	Kihon Checklist	24/79 (21.5%)	48.1%–30.4%–21.5%	-	The CAT score was higher in frail COPD group (15.2 ± 9.1) group compared to pre-frail (7.3 ± 5.6) and non-frail (5.8 ± 4.7) COPD group (*p* < 0.05).
Ierodiakonou D et al., 2019 Greece [43]	257 patients from primary care (cross-sectional study) with diagnosis of COPD according to GOLD guidelines; complete data on frailty in 253 patients	No age cut-off 65 ± 12.3	FiND Questionnaire	208/253 (82%)	18%–NA–82%	The mMRC values were higher in frail COPD group (2.16 ± 1.1) versus non-frail group (0.91 ± 0.8) (*p* < 0.001).	The CAT score was higher in frail COPD group (18.27 ± 6.4) versus non-frail group (13.86 ± 5.9) (*p* = 0.002).

mMRC dyspnoea scale = modified Medical Research Council dyspnoea scale. CAT = COPD Assessment Test. ^£^ values expressed as mean or median (IQR).

## Data Availability

Not applicable.
